# HLA-A alleles including HLA-A29 affect the composition of the gut microbiome: a potential clue to the pathogenesis of birdshot retinochoroidopathy

**DOI:** 10.1038/s41598-020-74751-0

**Published:** 2020-10-19

**Authors:** Peter R. Sternes, Tammy M. Martin, Michael Paley, Sarah Diamond, Mark J. Asquith, Matthew A. Brown, James T. Rosenbaum

**Affiliations:** 1 Australian Translational Genomics Centre, Institute of Health and Biomedical Innovation At Translational Research Institute, Queensland University of Technology, Brisbane, Australia; 2 Department of Ophthalmology, Casey Eye Institute, Oregon Health & Science University, Portland, OR USA; 3 Department of Medicine, Washington University, St. Louis, MO USA; 4 Department of Medicine, Oregon Health & Science University, Portland, OR USA; 5 Guy’s and St Thomas’ NHS Foundation Trust and King’s College London NIHR Biomedical Research Centre, London, USA; 6 Legacy Devers Eye Institute, Portland, OR USA

**Keywords:** Microbiome, Autoimmune diseases

## Abstract

Birdshot retinochoroidopathy occurs exclusively in individuals who are *HLA-A29* positive. The mechanism to account for this association is unknown. The gut microbiome has been causally implicated in many immune-mediated diseases. We hypothesized that *HLA-A29* would affect the composition of the gut microbiome, leading to a dysbiosis and immune-mediated eye disease. Fecal and intestinal biopsy samples were obtained from 107 healthy individuals from Portland, Oregon environs, 10 of whom were *HLA-A29* positive, undergoing routine colonoscopy. Bacterial profiling was achieved via 16S rRNA metabarcoding. Publicly available whole meta-genome sequencing data from the Human Microbiome Project (HMP), consisting of 298 healthy controls mostly of US origin, were also interrogated. PERMANOVA and sparse partial least squares discriminant analysis (sPLSDA) demonstrated that subjects who were *HLA-A29* positive differed in bacterial species composition (beta diversity) compared to *HLA-A29* negative subjects in both the Portland (p = 0.019) and HMP cohorts (p = 0.0002). The Portland and HMP cohorts evidenced different subsets of bacterial species associated with *HLA-A29* status, likely due to differences in the metagenomic techniques employed. The functional composition of the HMP cohort did not differ overall (p = 0.14) between *HLA-A29* positive and negative subjects, although some distinct pathways such as heparan sulfate biosynthesis showed differences. As we and others have shown for various HLA alleles, the HLA allotype impacts the composition of the microbiome. We hypothesize that *HLA-A29* may predispose chorioretinitis via an altered gut microbiome.

## Introduction

HLA molecules affect the susceptibility to at least 100 diseases^1,2^. Although HLA molecules play an essential role in antigen presentation, in most instances, the mechanisms by which HLA molecules predispose to disease are unknown. One hypothesis is that HLA molecules could affect disease susceptibility indirectly by shaping the composition of the gut microbiome^3,4^. The microbiome plays a major role in exposing the immune system to a wide range of antigens and thus educating it^4,5^. A teleological argument is that the tremendous polymorphism of the major histocompatibility system minimizes the risk that an infectious pathogen could eliminate all members of a species. We and others have shown that indeed one’s HLA type has an impact on which bacteria are present in the gut^3,6–10^. For example, *HLA-B27* which predisposes to ankylosing spondylitis and *HLA-DRB1* alleles associated with rheumatoid arthritis each have effects on the intestinal microbiome^3^.

Birdshot retinochoroidopathy (BSRC) is a vision-threatening, immune-mediated inflammation of the posterior uveal tract^11^. It occurs exclusively in individuals who have the HLA allele, A29^12–15^. *HLA-A29* might increase the risk to develop BSRC more than 200-fold^16^. We hypothesized that the effect of *HLA-A29* on disease susceptibility might be an indirect mechanism through an alteration of the gut microbiome. We tested this hypothesis by characterizing the microbiome in *HLA-A29* healthy individuals compared to *HLA-A29* negative controls.

## Methods

### Human participants

This study utilized collected human samples collected in previous studies. As described by Asquith et al. 2019^3^, the Portland cohort consisted of 107 subjects, aged 40–75, predominately Caucasian (~ 90%), typically following an omnivorous diet (~ 95%) and were undergoing routine colorectal cancer screening at Oregon Health & Science University’s Center for Health and Healing were included in this study. Individuals were excluded if they had a personal history of inflammatory bowel disease or colon cancer, prior bowel or intestinal surgery or were pregnant. Ethical approval for this study was obtained from the Oregon Health & Science University Institutional Review Board (IRB). Written informed consent was obtained from all subjects. This study was performed in accordance with all applicable US federal and state regulations, following the tenants of the Declaration of Helsinki, are as previously described^3^.

For the Human Microbiome Project (HMP) cohort, sample collection, storage, handling, and whole-metagenome sequencing were performed as in the HMP1^17^. Minimally perturbed microbiomes from 149 men and 151 women, mean age 26 years, mean BMI 24 kg/m^2^ and predominately Caucasian with strict inclusion and exclusion criteria (such as presence of systemic diseases, use of immunomodulators and recent use of antibiotics or probiotics) were sampled from multiple body sites at either two or three timepoints. Approval for this study was granted by the Institutional Review Boards of the two recruitment centers (Baylor College of Medicine, Houston, TX and Washington University, St. Louis, MO) and informed written consent was attained. Further details on IRB review, informed consent, subject exclusion criteria, the sampling protocols, and timeline can be found in previous publications^2,17,18^.

### Sample and data processing

For the Portland cohort, 568 stool and biopsy samples across 107 individuals were extracted and amplified for the bacterial marker gene 16S rRNA, as previously described^3^. Paired end reads were joined, quality filtered and analysed using Quantitative Insights Into Microbial Ecology (QIIME) v1.9.1^19^. Operational taxonomy units (OTU) were picked against a closed reference and taxonomy was assigned using the Greengenes database (gg_13_8)^20^, clustered at 97% similarity by UCLUST^21^ and low abundance OTUs were removed (< 0.01%). Genotyping was performed on DNA extracted from mucosal biopsies and genotyped using Illumina CoreExome SNP microarrays according to standard protocols. Bead intensity data were processed and normalized for each sample, and genotypes called using Genome Studio (Illumina). We imputed HLA-A genotypes using SNP2HLA^22^, as previously reported^23^.

For the HMP cohort, metadata were collected with permission from the Database of Genotypes and Phenotypes (dbGaP; https://www.ncbi.nlm.nih.gov/gap) with the accession number phs000228.v4.p1. Precomputed metagenomic profiles and basic subject metadata were collected through the HMP Data Analysis and Coordination Center (HMP DACC; https://hmpdacc.org), as described by Lloyd-Price et al. 2017^24^.

### Data visualization and statistical analysis

Multidimensional data visualisation of beta diversity was conducted using a sparse partial least squares discriminant analysis (sPLSDA) on arcsine squared root transformed data, as implemented in R v3.5.2^25^ as part of the MixOmics package v6.3.1^26^. Association of the microbial composition (beta diversity) with metadata of interest was conducted using a PERMANOVA test as part of vegan v2.4–5^27^ on arcsine square root transformed data at species level, taking into account individual identity where multiple sites per individual were co-analysed, as well as the sources of covariation such as BMI, ethnicity, age and gender. Alpha diversity was calculated at species level using the rarefy function as implemented in vegan v2.4–5 and differences were evaluated using a Wilcoxon rank-sum test. Differential abundance of bacterial taxa and MetaCyc pathways were tested for significance using MaAsLin2 v0.2.3^28^. Graphs and figures were generated using ggplot2 v3.3.2^29^.

## Results

Recently, we demonstrated that carriage of the main risk alleles for ankylosing spondylitis and rheumatoid arthritis (*HLA-B27* and *HLA-DRB1* risk alleles, respectively) correlated with microbiome perturbance in healthy individuals^3^. It follows that carriage of *HLA-A29*, the main risk allele for BSRC, may also be correlated with such an effect. To investigatee this, we analyzed two independent cohorts of healthy individuals, (1) a cohort of 107 healthy individuals with samples from six body sites (a.k.a. the Portland Cohort) analyzed via 16S rRNA metabarcoding, and (2) a cohort of 298 healthy individuals who have had their stools whole-metagenome sequenced as part of the Human Microbiome Project. Detailed descriptions of the cohorts can be found in their previous reports^2,17,18^ and a broad overview of the cohort according to *HLA-A29* status is shown in Table 1.

**Table 1:** Characteristics of the analyzed cohorts.

	HMP cohort			Portland cohort		
	*HLA-A29* Pos	*HLA-A29* Neg	p-value	*HLA-A29* Pos	*HLA-A29* Neg	p-value
Age (Mean ± SD)	25 ± 4.6	26 ± 5.2	0.37	55 ± 6.7	58 ± 7.4	0.3
BMI (Mean ± SD)	24.1 ± 3.6	24.34 ± 3.4	0.8	30.34 ± 8.8	28.23 ± 7.1	0.41
Gender (% Male)	25	53	0.02	40	46	0.7
Ethnicity (% White)	95	81	0.13	90	90	0.98
Count	21	277	–	10	97	–

Except for gender in the HMP cohort, age, BMI, gender and ethnicity were not significantly different between *HLA-A29* positive and negative groupings, as measured via T-test. All *HLA-A29* positive subjects carried the HLA-A29*02 subtype.

**Figure 1:**
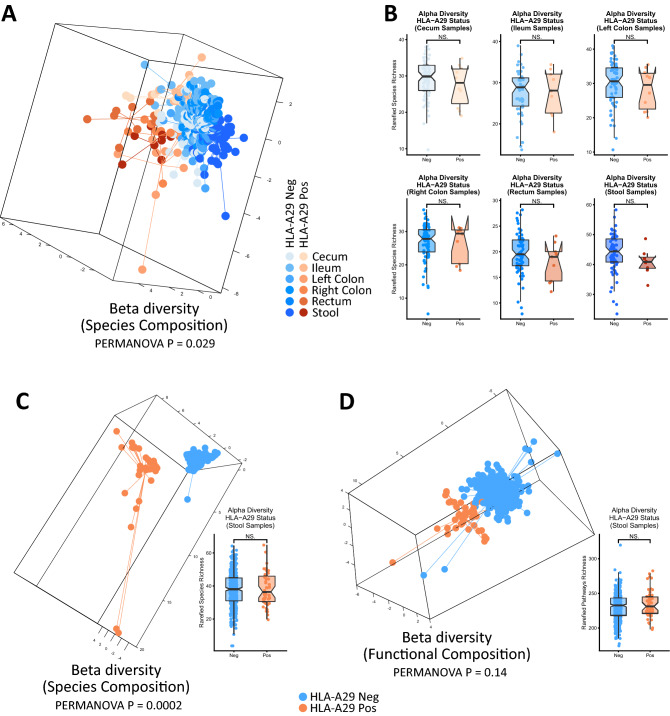
(**A**) sPLSDA and PERMANOVA analysis of bacterial species composition (beta diversity) of the Portland cohort. Controlling for covariates (gender, ethnicity, age and BMI) and accounting for repeated sampling, significant differentiation of the species composition according to *HLA-A29* status was noted. (**B**) Bacterial species richness (alpha diversity) across six sampling sites in the Portland cohort. Consistent with results noted for *HLA-B27* and *HLA-DRB1*, carriage of *HLA-A29* is not associated with a difference in the number of detectable bacterial species. (**C**) Alpha and beta diversity (sPLSDA and PERMANOVA) analysis of bacterial species (a.k.a. taxonomic) composition of the HMP cohort. Controlling for covariates (smoking, age, BMI, gender and ethnicity) and accounting for repeated sampling, significant differentiation was noted. No difference in alpha diversity was noted. (**D**) Alpha and beta diversity (sPLSDA and PERMANOVA) analysis of the overall metabolic/functional composition of the microbiome for individuals in HMP cohort. No significant differences were noted, consistent with observations that the functional composition of the microbiome is relatively stable across individuals compared to the taxonomic/species composition. Plots were generated using the mixOmics^26^ and ggplot2 packages as part of R.

Similar to our observations for *HLA-B27* and *HLA-DRB1*, we noted a significant differentiation of the overall bacterial species (a.k.a. taxonomic) composition in both the Portland (p = 0.019) and HMP (p = 0.0002) cohorts whilst accounting for sources of covariation such as age, gender, ethnicity and BMI. Furthermore, consistent with previous reports, no differences in the diversity of bacterial species was noted across both cohorts, indicating that the underlying host genetics may affect the overall composition of the microbiome, but not the overall species diversity (Fig. 1A–C). Of particular note was an observed enrichment of *Clostridium difficile* (q = 0.0007) for *HLA-A29* positive subjects in the HMP cohort (Supplementary Table 1). *Clostridium difficile* is well known for its ability to cause colitis and potentially associated immune responses to cause disease such as reactive arthritis^30^. The casual implications of *Clostridium difficile* enrichment and birdshot retinochoroidopathy may therefore warrant further investigation. Testing of other commonly found HLA alleles in the HMP dataset (> 5% allele frequency; *-A1*, *-A2*, *-A3*, *-A11*, *-A24*, *-A26* and *-A68*) also revealed significant differentiation of the overall taxonomic composition (beta diversity, p < 0.01), however the individual bacterial species associated with these various HLA-A subtypes were different, in particular no association with *Clostridium difficile* was found (Supplementary Table 3)*.* These data support allele-specific effects for HLA-A upon the microbiome.

Interrogation of the metabolic/functional composition, enabled by whole-metagenome sequencing information from the HMP cohort, did not reveal significant differentiation of the overall composition (beta diversity) nor diversity (alpha diversity) (Fig. 1D). Despite no differences in the overall composition, some specific metabolic pathways were significantly different (Supplementary Table 2), notably a potential enrichment of Heparan sulfate biosynthesis pathways (q = 0.07).

## Discussion

Although HLA molecules are responsible for antigen presentation, the mechanism by which HLA molecules predispose to disease is most often unknown. HLA molecules influence susceptibility in some immune-mediated diseases such as ankylosing spondylitis^3^, BSRC^12^, and Crohn’s disease^31^ in which auto-antibodies are not a characteristic finding in the disease. This would suggest that in such diseases, the predisposition engendered by the HLA allele might not be acting via an autoimmune response. Since the gut microbiome educates the immune response and includes a vast array of antigenic diversity, an alternative hypothesis is that HLA molecules in some instances predispose to disease via effects on the gut microbiome^4^. An alteration in the gut microbiome could affect disease susceptibility through a variety of mechanisms such as a change in the balance of T cell subsets, migration of lymphocytes from the gut to an affected organ, dysbiosis leading to bacterial antigens leaking from the gut^32^, or molecular mimicry^4^. The vast majority of individuals who are *HLA-A29* positive, do not develop birdshot retinochoroidopathy^33^. Likewise, the majority of individuals who are *HLA-B27* positive do not develop spondyloarthritis and the majority of individuals who are *HLA-DRB1* positive do not develop rheumatoid arthritis. Whilst HLA alleles are major susceptibility factors for many immune-mediated inflammatory diseases, many other genetic loci have been associated with disease, notably *ERAP1* and *ERAP2* in the case of BSRC^34,35^. Carriage of these risk alleles is necessary, but not sufficient, for disease which suggests that the presence of other environmental or genetic factors is also required. A notable example of this is celiac disease, for which 30–35% of the population carry the *HLA-DQ2* and *-DQ8* risk alleles, yet only ~ 2–5% of the carriers develop disease. Additional non-major histocompatibility complex risk loci have been identified, however their overall genetic contribution has been estimated as only 3–4%. Environmental factors such as enteropathic virus and changes in bacterial flora have subsequently been shown to favor the development of celiac disease^36^.

In this report, we interrogated two separate databases, one derived from the Human Microbiome Project^37^, and one obtained in Portland, Oregon, USA from healthy subjects undergoing colonoscopy^3^. In both instances, we found that *HLA-A29* affected the bacterial composition of the intestine. We did not compare the effect of HLA-A*2901 versus A*2902 since no HLA-A*2901 individuals were found in the analyzed cohorts. Whilst both databases showed microbiome differences in *HLA-A29* carriers, differences in findings for specific bacterial species occurred between the databases. However, in this circumstance, direct inter-cohort comparison is not entirely valid given the differences in cohort demographics (such as age and BMI) as well as statistical power and methodological differences, as the HMP data are based on whole-metagenome sequencing while the Portland data are based on 16S metabarcoding. There is precedent to indicate that these different methodologies produce alternative results^38–40^. However, even though the abundance of specific bacterial species cannot be directly compared between cohorts, re-confirmation of the broader net effect on taxonomic composition in an independent cohort remains possible.

In this study we elected to test the effect of *HLA-A29* on the intestinal microbiome in healthy individuals. A future study should compare the intestinal microbiome in patients with BSRC with healthy, *HLA-A29* positive individuals or individuals with other forms of posterior uveitis. The rationale to study healthy subjects is that it allows us to analyze specifically the single, most important known risk factor for this form of uveitis. Our data offer a plausible mechanism by which *HLA-A29* might contribute to the disease. If the intestinal microbiome in patients with BSRC does not differ from the microbiome of healthy*, HLA-A29* subjects, the other genetic factors or environmental factors must contribute via a mechanism that does not involve the microbiome. If BSRC patients, on the other hand, have an intestinal microbiome that differs from healthy, *HLA-A29* controls, a reasonable hypothesis is that additional causal factors also contribute to the predisposition to BSRC via an effect on the microbiome.

We recognize that demonstrating that *HLA-A29* shapes the microbiome does not prove that the changes in the microbiome are causally related to the uveitis. The pathogenesis of BSRC, however, remains unknown. The potential contribution of the gut microbiome suggests novel approaches in human and animal models to elucidate the cause of this vision-threatening disease.

## Supplementary information

Supplementary file1
